# RNase H2 Loss in Murine Astrocytes Results in Cellular Defects Reminiscent of Nucleic Acid-Mediated Autoinflammation

**DOI:** 10.3389/fimmu.2018.00587

**Published:** 2018-03-29

**Authors:** Kareen Bartsch, Markus Damme, Tommy Regen, Lore Becker, Lillian Garrett, Sabine M Hölter, Katharina Knittler, Christopher Borowski, Ari Waisman, Markus Glatzel, Helmut Fuchs, Valerie Gailus-Durner, Martin Hrabe de Angelis, Björn Rabe

**Affiliations:** ^1^Medical Faculty, Institute of Biochemistry, Christian-Albrechts-University Kiel, Kiel, Germany; ^2^Institute for Molecular Medicine, University Medical Center of the Johannes Gutenberg University Mainz, Mainz, Germany; ^3^German Mouse Clinic, Institute of Experimental Genetics, Helmholtz Zentrum München, German Research Center for Environmental Health, Neuherberg, Germany; ^4^Institute of Developmental Genetics, Helmholtz Zentrum München, German Research Center for Environmental Health, Neuherberg, Germany; ^5^Institute of Neuropathology, University Medical Center Hamburg-Eppendorf, Hamburg, Germany; ^6^Chair of Experimental Genetics, School of Life Science Weihenstephan, Technische Universität München, Freising, Germany; ^7^German Center for Diabetes Research (DZD), Neuherberg, Germany

**Keywords:** Aicardi–Goutières syndrome, RNase H2, interferon signature, DNA damage, cellular senescence

## Abstract

Aicardi–Goutières syndrome (AGS) is a rare early onset childhood encephalopathy caused by persistent neuroinflammation of autoimmune origin. AGS is a genetic disorder and >50% of affected individuals bear hypomorphic mutations in ribonuclease H2 (RNase H2). All available RNase H2 mouse models so far fail to mimic the prominent CNS involvement seen in AGS. To establish a mouse model recapitulating the human disease, we deleted RNase H2 specifically in the brain, the most severely affected organ in AGS. Although RNase H2^ΔGFAP^ mice lacked the nuclease in astrocytes and a majority of neurons, no disease signs were apparent in these animals. We additionally confirmed these results in a second, neuron-specific RNase H2 knockout mouse line. However, when astrocytes were isolated from brains of RNase H2^ΔGFAP^ mice and cultured under mitogenic conditions, they showed signs of DNA damage and premature senescence. Enhanced expression of interferon-stimulated genes (ISGs) represents the most reliable AGS biomarker. Importantly, primary RNase H2^ΔGFAP^ astrocytes displayed significantly increased ISG transcript levels, which we failed to detect in *in vivo* in brains of RNase H2^ΔGFAP^ mice. Isolated astrocytes primed by DNA damage, including RNase H2-deficiency, exhibited a heightened innate immune response when exposed to bacterial or viral antigens. Taken together, we established a valid cellular AGS model that utilizes the very cell type responsible for disease pathology, the astrocyte, and phenocopies major molecular defects observed in AGS patient cells.

## Introduction

Aicardi–Goutières syndrome (AGS) is an inherited autoimmune disorder typically affecting the brain and, to a lesser extent, also the skin. Its neurological symptoms include leukodystrophy, microcephaly, and calcifications within the basal ganglia and white matter (1). Disease onset is typically during the first year of life, although in about 20% disease symptoms manifest already at birth or *in utero*. In the affected neonates, clinical signs of AGS mimic the sequelae of congenital virus infections. AGS is caused by mutations in the genes *TREX1, RNASEH2, SAMHD1, ADAR1*, or *IFIH1*, with RNase H2 mutations accounting for >50% of all cases (2). RNase H2 is a trimeric enzyme consisting of the subunits A (catalytic), B, and C, whereby all three subunits can be mutated, although individually, in AGS patients. With the exception of IFIH1, which encodes the cytosolic RNA sensor MDA5, all proteins associated with AGS are involved in nucleic acid metabolism. It has therefore been proposed that in AGS endogenous nucleic acids accumulate, thereby stimulating the innate immune system to produce increased amounts of the antiviral cytokine interferon α (3). While elevated interferon α levels are found in AGS patients in the cerebrospinal fluid and to a lesser extent also in serum, mRNA expression of interferon-stimulated genes (ISGs) in peripheral blood has turned out to be the most reliable AGS biomarker to date (4). This so-called interferon signature is also a hallmark of systemic lupus erythematosus (SLE), which shares many clinical features with AGS (3, 5). Post-mortem analysis of diseased brains has established astrocytes as the cellular source of interferon α in AGS (6). In line with this, transgenic mice that constitutively express interferon α in astrocytes develop neurological symptoms similar to AGS, suggesting that AGS is driven by chronic cerebral presence of interferon α (7). A striking feature of all previous AGS mouse models is that none seems to recapitulate the strong CNS involvement associated with the human disease. TREX1 knockout (KO) mice for instance exhibit multi-organ autoimmunity and eventually succumb to sterile myocarditis, which is accompanied by strong stimulation of the type I interferon axis mainly in the heart but also in other tissues. The brain, however, is not affected in TREX1 KO mice (8, 9). Both ADAR1 and RNase H2 KO mice die during embryogenesis, with only ADAR1 KO embryos displaying marked type I interferon upregulation (10–12). SAMHD1-deficient mice on the other hand have no obvious phenotype, but exhibit a weak interferon signature in selected organs (13, 14). Mice bearing a gain-of-function IFIH1 mutation show lupus-like systemic autoimmunity, again without CNS involvement (15). Two hypomorphic RNase H2 mouse models have been generated recently and depending on the introduced patient mutation, the animals die perinatally (G37S mutation in the catalytic A subunit) or display no overt phenotype (A177T mutation in the accessory B subunit) (16, 17). Although both mouse models exhibit ISG upregulation in some tissues, no signs of neuroinflammation were apparent. The differences in disease presentation in terms of CNS involvement between AGS patients and the corresponding mouse models are striking, yet the underlying reasons are unclear. Given the absence of a CNS-related phenotype in hypomorphic RNase H2 mice and the early embryonic lethality of RNase H2 null mice, we generated conditional knockout mice devoid of RNase H2 in large parts of the brain (RNase H2^ΔGFAP^ mice) in an attempt to establish an RNase H2-based mouse model of AGS. In contrast to the severe phenotype associated with human disease, but in line with other AGS models, mice lacking RNase H2 in astrocytes and neurons displayed no clinical signs of disease. However, astrocytes isolated from RNase H2^ΔGFAP^ mice exhibited cellular defects reminiscent of AGS. We believe that the RNase H2-deficient astrocytes generated in this study can serve as a valid cellular model for AGS, especially as they represent the very cell type considered to govern the pathogenic interferon α expression in human patients.

## Materials and Methods

### Mice

RNase H2Bflox mice, the generation of which was described in Ref. (11), were kindly provided by Axel Roers (Institute of Immunology, Medical Faculty Carl Gustav Carus, University of Technology Dresden, Dresden, Germany). Floxed RNase H2B mice were crossed to Cre-hGFAP mice (18) for gene inactivation in astrocytes as well as cortical and hippocampal neurons and resulting RNase H2B^flox/flox^; hGFAP-Cre mice were termed RNase H2^ΔGFAP^ mice. For RNase H2 ablation in neurons of the neocortex and hippocampus floxed RNase H2B mice were intercrossed with Cre-Emx1 mice (19) and RNase H2B^flox/flox^; Emx1-Cre mice were termed RNase H2^ΔEmx1^ mice. RNase H2B^flox/flox^ littermates w/o Cre served as control animals. All mice were provided with food and water *ad libitum* and maintained in a 12-h light–dark cycle under standard conditions at Kiel University. Genotyping PCR was performed on processed tail clips (Direct PCR Lysis Reagent Tail, Peqlab), whereby respective PCR protocols as well as primer sequences were obtained from www.jax.org. Procedures involving animal care were conducted in agreement with national and international laws and policies with appropriate permission. All experiments were carried out in accordance with the guidelines for Animal Care of the Christian-Albrechts-University of Kiel.

### Generation and Treatment of Primary Astrocytes

For astrocyte isolation, P3/4 pups were decapitated, their brains taken out, and meninges carefully removed under a dissecting microscope. Cerebral hemispheres were minced with a scalpel and digested in HBSS complemented with 2.5% trypsin (v/v) and 1% DNase I (m/v) for 30 min at 37°C (water bath). During digestion, the suspension was disintegrated every 10 min using a 1 ml pipet. Afterwards the cell suspension was filtrated through a 40 µm cell strainer and the filtrate topped up with two volumes minimum essential medium (MEM). Following centrifugation for 3 min at 1,300 *g*, cells were resuspended in glia medium 1 (MEM complemented with 10% horse serum, 0.6% glucose, and 1% penicillin/streptomycin) and dispensed into three 6-wells coated with 0.1 mg/ml poly-l-lysine. After 48 h, culture medium was changed to glia medium 2 (Dulbecco’s modified Eagle’s Medium, high Glucose (4.5 g/l), with GlutaMAX™, supplemented with 10% FCS, N2 Supplement, 1 μg/50 ml epidermal growth factor, and 1% penicillin/streptomycin) in which cells were cultured at 37°C, 5% CO_2_ and 3% O_2_ until experimental use. Upon confluency, control and RNase H2^ΔGFAP^ astrocytes were splitted 1:6 and 1:2, respectively, using Accutase and Trypsin/EDTA (2:3, Sigma Aldrich). A day prior to experiments microglia were removed from cell suspensions by multiple thorough PBS washing steps. The primary astrocyte culture consistently contained >90% GFAP-positive cells. Astrocytes were treated with Doxorubicin (1 µM for 18 h) and LPS (*E. coli* 0111:B4, 500 ng/ml, 6 h, both Sigma Aldrich) or transfected with polyI:C (5 µg, 6 h, Invivogen). To drive astrocytes into oncogene-induced senescence (OIS), cells were retrovirally transduced with H-Ras^G12V^ 4 days post isolation, as described in Ref. (20). Successful transfectants were enriched by 500 µg/ml puromycin (Sigma Aldrich) treatment for another 6 days and senescent astrocytes were harvested and analyzed 10 days post isolation.

### Immunofluorescence and Immunohistochemistry

For immunofluorescence analysis, primary astrocytes were fixed with ice-cold acetone/methanol (1:2) for 10 min and blocked with 1% (w/v) BSA in PBS overnight at 4°C. Cells were incubated with primary antibodies for 4 h and secondary antibodies for 2 h in a humidified chamber and subsequently embedded in Immu-Mount (Thermo Fisher Scientific) complemented with 5 µg/ml 4′,6-diamino-2-phenylindole (DAPI, Sigma Aldrich). Anti-histone γH2AX (Cell Signaling, #2577) and rabbit anti-mouse RNase H2 antiserum [kindly provided by Andrew P. Jackson, MRC Human Genetics Unit, Institute of Genetics and Molecular Medicine, University of Edinburgh, UK, described in Ref. (12)] were used as primary antibodies, while anti-rabbit Cy3 (GE Healthcare) served as secondary Ab. For immunohistochemistry, mice were perfused transcardially *via* the left ventricle with PBS followed by 4% PFA (in PBS). Brains were removed and post-fixed additional 4 h by immersion at room temperature. Subsequently, brains were transferred to 30% sucrose (in PBS) overnight. 35 µm thick free floating sections were prepared on a Leica 9,000 s microtome (Leica, Wetzlar, Germany). Sections for immunohistochemistry were blocked with 1.6% H_2_O_2_ solution and washed in PBS. Sections for immunohistochemistry and immunofluorescence were rinsed in PBS and subsequently blocked with 4% normal goat serum in PBS containing 0.2% bovine serum albumin and 0.25% Triton X-100 (blocking solution) at room temperature for 2 h. Primary antibodies (anti-GFAP, clone G-A-5, Sigma; Ki-67, SolA15, eBioscience; anti-Iba-1, GTX100042, Genetex; anti-NeuN, MAB377, Millipore) were diluted in blocking solution and sections were incubated overnight at 4°C with gentle agitation. After washing 3× with washing buffer (PBS containing 0.25% TX-100), sections were incubated for 2 h at RT with appropriate secondary antibodies [fluorophore conjugated; AlexaFluor 488 and 594 (Molecular Probes) or biotinylated second antibodies (Vector Laboratories)] diluted in washing buffer. Antibody dilutions are provided in Supplementary Table B. After additional three washes with washing buffer, sections were coverslipped with MOWIOL/DABCO (fluorophore-labeled sections) containing DAPI or incubated with Streptavidin-HRP (ABC Elite Kit, Vector Laboratories) for another 1 h at room temperature followed by another three washes, development with 3,3′-Diaminobenzidin substrate solution, and coverslipped in MOWIOL. A FV1000D Laser Scanning Confocal Microscope (Olympus) equipped with FV10-ASW 4.2 Viewer software was used for image acquisition.

### qPCR, Western Blotting, and FACS

Total RNA was isolated using the Gene Jet RNA Purification Kit (Thermo Fisher Scientific), while cDNA was generated with Revert Aid Reverse Transcriptase (Thermo Fisher Scientific) according to the manufacturer’s instructions, except a mixture of oligo(dT)18 and random hexamer primers (1:2, Thermo Fisher Scientific) was used. The qPCR reaction contained SYBR Green qPCR Master Mix (Thermo Fisher Scientific), 0.5 µM target specific primer pairs (Table A in Supplementary Material), and 10 ng cDNA template. SYBR Green incorporation was analyzed on a Lightcycler 480 II (Roche Applied Science) according to the following program: initial denaturation at 95°C for 10 min, 45 cycles of denaturation (10 s at 95°C), annealing (20 s at 60°C), and elongation (20 s at 72°C). Relative mRNA expression was calculated based on the ΔΔCp method with glyceraldehyde 3-phosphate dehydrogenase (GAPDH) serving as house keeping control. For flow cytometry, detached astrocytes were fixed in 4% PFA (in PBS) for 20 min, saponin-permeabilized for 15 min (using *saponin-based permeabilization and wash reagent*, Invitrogen), and blocked in 1% BSA (in PBS) for 15 min Afterward, cells were incubated with FITC-coupled anti-histone γH2AX (Cell Signaling, #9719) for 1 h on ice and resulting fluorescence was detected using a FACSCanto flow cytometry system (BD biosciences). Western Blot analysis was performed as described in Ref. (21) and the following primary antibodies were used: anti-LAMP1 (DSHB, 1D4Bc), anti-RNase H2A (Origene, TA306706), anti-H-Ras (Santa Cruz, sc-29), and anti-PARP1 (Cell Signaling, #9542). To ensure equal loading amounts, membranes were stripped and re-probed with anti β-actin (Cell Signaling Technology, #4967). Antibody dilutions are provided in Supplementary Table B.

### Neurological and Behavioral Analyses

The neurological as well as behavioral tests were performed at the German Mouse Clinic (22, 23). The test pipeline started with 39 mice (12 male mutants, 7 male controls, 10 female mutants, and 10 female controls). Mice were maintained in IVC cages with water and standard mouse chow (Altromin no. 1314) according to the GMC housing conditions and German laws (for details, check https://www.mouseclinic.de/about-gmc/mouse-husbandry/index.html). All tests performed at the GMC were approved by the responsible authority of the Regierung von Oberbayern (animal license no. 46-16). Welfare-related monitoring of experimental animals was performed according to the animal license. Sample size of the experiments was calculated and documented in the approval 46-16.

#### Open Field

The Open Field analysis was carried out as described previously (24, 25). It consisted of a transparent and infra-red light permeable acrylic test arena with a smooth floor (internal measurements: 45.5 cm × 45.5 cm × 39.5 cm). Illumination levels were set at approx. 150 lux in the corners and 200 lux in the middle of the test arena. Data were recorded and analyzed using the ActiMot system (TSE, Bad Homburg, Germany).

#### Prepulse Inhibition (PPI) of the Acoustic Startle Response

Animals were separated based on sex, but not genotype. PPI was assessed automatically using an automated startle apparatus setup (Med Associates Inc., VT, USA) including four identical sound-attenuating cubicles. The protocols were written using the Med Associates “Advanced Startle” software. Experiments were carried out between 08:30 h and 17:00 h. Background noise was 65 dB, and startle pulses were bursts of white noise (40 ms). A session was initiated with a 5-min-acclimation period followed by five presentations of leader startle pulses (110 dB) that were excluded from statistical analysis. Trial types for the PPI included four different prepulse intensities (67, 69, 73, 81 dB); each prepulse preceded the startle pulse (110 dB) by a 50 ms inter-stimulus interval. Each trial type was presented 10 times in random order, organized in 10 blocks, each trial type occurring once per block. Inter-trial intervals varied from 20 to 30 s. This protocol is based on the protocol used in IMPRESS from the International Mouse Phenotyping Consortium (IMPC, see www.mousephenotype.org/impress), adapted to the specifications of our startle equipment.

#### Y Maze

Spontaneous alternations were assessed using the Y-Maze, which was made of opaque light gray PVC and had three identical arms (30 cm × 5 cm × 15 cm) placed at 120° from each other; illumination in the center of the maze was 100 lux. Each mouse was placed at the end of one arm and allowed to move freely through the maze during a 5-min session. Spontaneous alternations (defined as consecutive entries into all three arms without repetitions in overlapping triplet sets) were scored. Total numbers of arm entries were collected cumulatively over the 5 min. Spontaneous alternation performance percentage is defined as the ratio of actual (total alternations) to possible alternations (total number of triplets) × 100. When placed in the Y-Maze, normal mice prefer to explore the least recently visited arm, and thus tend to alternate visits between the three arms. To explore the three arms successively the mouse must maintain an ongoing record of the most recently visited arms, and continuously update such records. Therefore, alternation behavior is a measure of spatial working memory.

#### Social Discrimination

The Social Discrimination procedure consisted of two 4-min exposures of stimulus animals (ovariectomized 129Sv females) to the test animal in a fresh cage to which the test animal had been moved 2 h prior to testing. All stimulus animals are identified using colored non-toxic non-permanent paint markers on the tail. After a retention interval of 2 h, this stimulus animal was re-exposed to the test animal together with an additional, previously not presented stimulus animal. A separate “familiar” and “unfamiliar” stimulus animal was assigned to each test animal. The duration of investigatory behavior of the test animal toward the stimulus animals (familiar and unfamiliar) during this test phase was again recorded by a trained observer with a hand-held computer. A social recognition index was calculated as time spent investigating the unfamiliar stimulus mouse/time spent investigating both the familiar and unfamiliar stimulus mouse.

#### Beam Walk

Motor skills were tested on beams with different diameters (20 and 12 mm square and 22 and 15 mm round). The traversing time and numbers of falls, foot slips, and stops were recorded. The mouse performed three trials each consecutively, and the average time of these three trials was calculated.

#### Beam Ladder

The beam ladder consists of two Plexiglas screens connected with several metal beams of variable distance. The test is used to evaluate skilled walking of the mice. Mice traverse the ladder and foot slips of fore paws and hind paws are counted separately as well as traversing time.

Modified SHIRPA analysis, rotarod, auditory brain stem response (ABR), grip strength, and hot plate testing was performed as described (26).

### Experimental Autoimmune Encephalomyelitis (EAE)

Mice were immunized subcutaneously at the tail basis with 50 µg MOG_35–55_ peptide (Gene Script) emulsified in Complete Freund’s adjuvant (Difco) supplemented with 1.1 mg of heat-inactivated *Mycobacterium tuberculosis* (Difco). At the day of immunization and again 2 days later, mice received an intraperitoneal injection of 200 ng pertussis toxin (List Biological Laboratories). Clinical signs of disease were assessed daily and graded on a scale from 0 to 6 as follows: 0, no disease; 0.5, partial loss of tail tonicity; 1, complete loss of tail tonicity; 1.5, partially impaired righting reflex on attempt to roll over (within 3 s); 2, impaired righting reflex; 2.5 partial hind limb paresis resulted in staggering gait; 3, complete hind limb paresis; 3.5, complete hind limb paralysis; 4, unilateral forelimb paresis; 4.5, complete forelimb paresis; 5, moribund, and 6, dead animal. Experiments were performed in accordance with the guidelines of the central animal facility institution (TARC, University of Mainz) and approved by the local authorities under the license number G 13-1-098.

### Statistics

All data are shown as mean ± SEM. Statistical analysis was performed with GraphPad Prism 5 using two-tailed Student’s *t*-test unless indicated otherwise in the figure legends. For all tests, *p*-values less than 0.05 were considered statistically significant. The phenotyping screen at the GMC was mainly designed as a high throughput screen for new phenotypic alterations, a correction for multiple testing of the various parameters was not performed. If not stated otherwise, data were analyzed using R software (Version 3.0.2; Foundation of Statistical Computing, Vienna, Austria).

## Results

### Generation of Conditional Knockout Mice Lacking RNase H2 in the Brain

**Figure 1:**
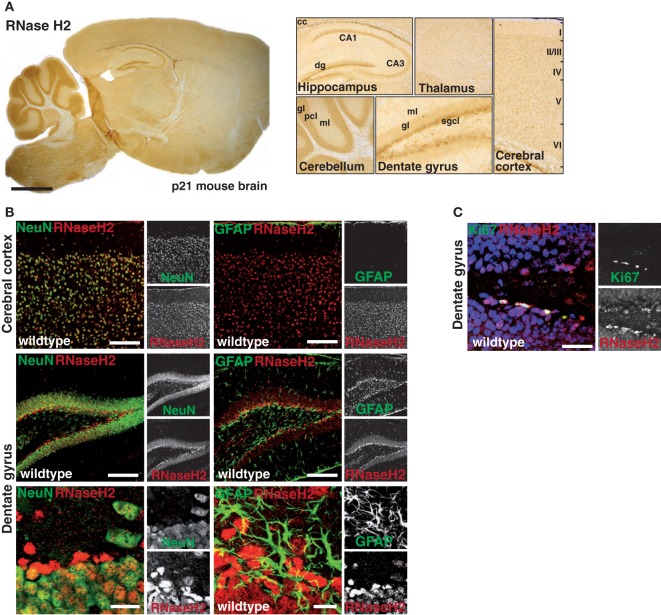
Expression of RNase H2 in the murine central nervous system. **(A)** Sagittal sections from p21 mouse brains were stained with an RNase H2-specific antiserum followed by a biotinylated secondary antibody, streptavidin-coupled HRP, and developed with the chromogenic substrate 3,3′-Diaminobenzidine (DAB). Note the high expression of RNase H2 in the corpus callosum (cc) and hippocampus, with particularly high expression in the subgranular cell layer (sgcl) of the dentate gyrus (dg). Cornu Ammoni (CA), molecular layer (ml), granular layer (gl). **(B)** Co-immunofluorescence staining for RNase H2 (red) with the neuronal marker NeuN (green, left panel) and astroglia marker GFAP (green, right panel) shows striking co-localization of RNaseH2 in neuronal cells expressing NeuN in both the cerebral cortex and dg. Scale bar = 150 µm (upper four panels), 10 µm (lower two panels) **(C)** Co-immunofluorescence staining for RNase H2 shows highest expression of RNase H2 in Ki67-positive proliferating cells. Scale bar = 30 µm.

**Figure 2:**
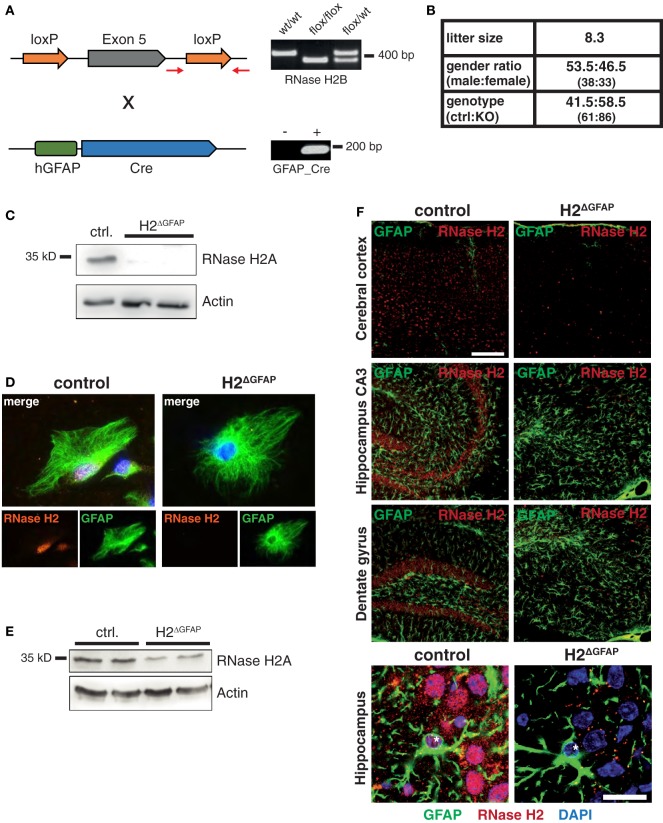
Validation of RNase H2^ΔGFAP^ conditional knockout mice. **(A)** Depiction of the relevant transgenic regions and RNase H2^ΔGFAP^ PCR genotyping strategy. Representative agarose gel images are shown on the right with respective genotypes above. Red arrows indicate PCR primer pair used for genotyping the floxed RNase H2B allele. **(B)** Breeding characteristics of RNase H2^ΔGFAP^ mice including litter size, gender, and genotype distribution. **(C)** Western Blot confirming loss of catalytic RNase H2A subunit in cultivated RNase H2^ΔGFAP^ astrocytes 7 days post isolation. β-actin served as loading control. **(D)** Representative confocal images showing lack of RNase H2 holo enzyme in RNase H2^ΔGFAP^ astrocytes 7 days post isolation. Astrocytes stained positive for GFAP, while DAPI was used as nuclear counter stain. **(E)** Immunoblot showing reduced RNase H2A protein levels in whole brain lysates of RNase H2^ΔGFAP^ mice (age 8 weeks, two mice per genotype). β-actin as loading control. **(F)** Absence of RNase H2 holoenzyme in the majority of cells in the cerebral cortex, hippocampus (CA3), and dentate gyrus of RNase H2^ΔGFAP^ mice (age 3 months). Astrocytes were co-stained with GFAP, while nuclei were counter-stained with DAPI. Note the absence of RNase H2 in both astrocytes (asterisk) and neuronal cells (lower panel). Scale bars = 150 µm.

As information regarding RNase H2 expression in the mammalian brain is scarce, we first tracked RNase H2 protein expression in the mouse brain using a rabbit antiserum raised against the RNase H2 holoenzyme. RNase H2 was expressed throughout the entire murine brain with highest expression in the hindbrain, the cerebellum, the hippocampus, and the cerebral cortex (Figure 1A). Notably, particularly high expression was observed in the subgranular zone (SGZ) of the hippocampal dentate gyrus (dg), a region typically associated with adult hippocampal neurogenesis and proliferation. To further narrow down RNase H2 expression, we applied double-immunofluorescence staining of RNase H2 with NeuN, a neuronal marker, and the astrocytic marker GFAP (Figure 1B). RNase H2 extensively co-localized with NeuN in both the cerebral cortex and the dg (Figure 1B). Remarkably, those cells with the highest expression in the SGZ were negative for GFAP and NeuN, a marker for more mature neurons, indicating that these cells were immature neuronal precursor cells (Figure 1B). Accordingly, these abundantly RNase H2 expressing cells were positive for Ki67, a marker for dividing cells (Figure 1C). Immunohistochemistry of diseased brain sections revealed that astrocytes secrete the pathogenic interferon α in AGS patients (6), and transgenic mice overexpressing interferon α from the supposedly astrocyte-specific GFAP-promoter exhibit clinical signs reminiscent of AGS (7). To establish a mouse model of AGS, we conditionally deleted RNase H2 in the mouse brain by intercrossing RNase H2B^flox^ mice with GFAP-Cre transgenic mice (Figure 2A). The resulting animals carrying homozygous RNase H2B deletions were designated RNase H2^ΔGFAP^ mice. RNase H2^ΔGFAP^ mice were viable, fertile, and exhibited no obvious phenotype. Offspring was generated in the expected Mendelian ratio (Figure 2B). Western Blot and immunofluorescence analysis revealed robust expression of RNase H2 in wild-type astrocytes, while astrocytes isolated from RNase H2^ΔGFAP^ mice completely lacked the nuclease (Figures 2C,D). Importantly, genetic ablation of RNase H2B has shown to be sufficient to disrupt expression of the entire holoenzyme (12). Absence of RNase H2 was confirmed *in vivo* by examining different brain regions using immunohistochemistry. RNase H2 was absent from the majority of cortical and hippocampal neurons as well as astrocytes in RNase H2^ΔGFAP^ mice (Figure 2F). In keeping with this, western blot analysis of whole brain lysates revealed a marked downregulation of RNase H2 protein expression (Figure 2E). Although leakiness of the technically astrocyte-specific GFAP-Cre line has been reported previously (27), we were surprised by the efficient ablation of RNase H2 in neuronal cells of RNase H2H2^ΔGFAP^ mice. To validate the prominent neuronal expression of RNase H2, we generated a second conditional RNase H2 knockout mouse line by crossing RNase H2B^flox^ mice to Emx1-Cre transgenic mice, in which Cre recombinase is active predominantly in neurons of the neocortex and hippocampus. Ablation of neuronal RNase H2 was effective in RNase H2H2^ΔEmxl^ mice and largely mirrored the deletion pattern seen in RNase H2^ΔGFAP^ mice (Figure S1A in Supplementary Material). However, because of the strong clinical link between astrocytes and AGS pathology, we focused in the present work on RNase H2^ΔGFAP^ animals.

### RNase H2Δ^GFAP^ Mice Display No AGS-Like Phenotype

**Figure 3:**
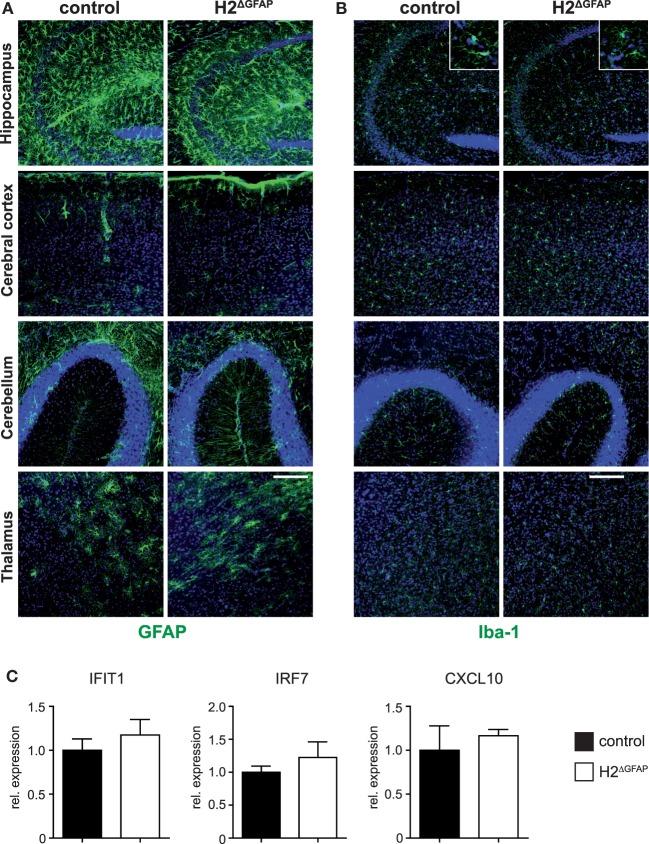
Absence of neuroinflammation in RNase H2^Δ^^GFAP^ mice. **(A)** Immunohistochemistry for the astrocyte marker GFAP reveals absence of significant astrogliosis in the hippocampus, cerebral cortex, cerebellum, and thalamus of RNase H2^ΔGFAP^ mice. Astrogliosis would present as increased expression of GFAP, cellular hypertrophy with changes of astrocyte morphology or proliferation of astrocytes. **(B)** Comparable Iba-1 staining between RNase H2^ΔGFAP^ and control brains indicates absence of microgliosis in RNase H2^ΔGFAP^ mice (age 3 months). Inserts represent magnifications of individual hippocampal microglia showing similar morphology in both genotypes. Scale bar = 150 µm. **(C)** Comparable mRNA expression of IFIT1, IRF7, and CXCL10 in RNase H2^ΔGFAP^ and control brains (*x* = 1), as quantified by qPCR. ISG expression was normalized to GAPDH. Whole brain RNA was prepared from P3-4 pups. Error bars are SEM, *t*-test (*n* = 5 brains/genotype).

Aicardi–Goutières syndrome is characterized by fundamental morphological changes of the brain, including microcephaly, cerebral calcifications, and white matter abnormalities such as atrophy (1). However, baseline H&E histology revealed no apparent differences between RNase H2^ΔGFAP^ and control brains (Figure S2A in Supplementary Material). Chronic neuroinflammation associated with activation of the type I interferon axis is a defining hallmark of AGS (2). Brains of 3 months old RNase H2^ΔGFAP^ mice did not show signs of astrogliosis or microgliosis compared to control littermates, as assessed by GFAP and Iba-1 immunostaining, respectively (Figures 3A,B). Importantly, qPCR analysis revealed similar expression of selected ISGs in RNase H2^ΔGFAP^ and control brains (Figure 3C). We additionally performed detailed neurological and behavioral tests with RNase H2^ΔGFAP^ animals (summarized in Figure S2B in Supplementary Material). However, behavioral and neurological examination of RNase H2^ΔGFAP^ mice only sporadically revealed subtle sex-specific effects of unclear relevance, which did not resemble the severe disabilities observed in AGS (summarized in Figure S2B, detailed in Figures S3A–F and Neurology Data in Supplementary Material). Disease progression in AGS patients with the same mutation often differs significantly, even within families (2). It has, therefore been suggested that external factors, such as viral infections or other environmental cues, may influence the course of disease or even trigger disease onset. To test this, we subjected mice to EAE, a mouse model of multiple sclerosis. However, following immunization with myelin oligodendrocyte glycoprotein peptide (MOG35-55), we observed no major differences in disease progression or clinical parameters between RNase H2^ΔGFAP^ and control mice (Figures S4A–C in Supplementary Material). In conclusion, RNase H2^ΔGFAP^ mice did not display AGS-related pathology and failed to show exacerbated disease upon induction of CNS autoimmunity. Furthermore, RNase H2^ΔEmx1^ mice developed normally and had no obvious phenotype. Similar to RNase H2^ΔGFAP^ mice, RNase H2^ΔEmx1^ animals lacked active astrogliosis or interferon signature (Figures S1A,B in Supplementary Material), resulting in a second brain-specific RNase H2 knockout mouse line that fails to recapitulate major signs of the human disease.

### Astrocytes Derived From RNase H2Δ^GFAP^ Mice Show Cellular Defects Consistent With Aicardi–Goutières Syndrome-Like Autoinflammation

**Figure 4:**
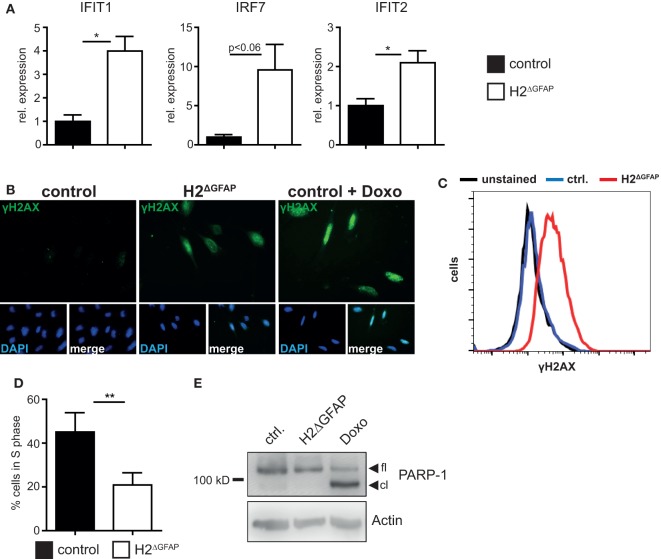
RNase H2^ΔGFAP^ astrocytes recapitulate features of Aicardi–Goutières syndrome patient cells. **(A)** Increased expression of selected interferon-stimulated genes (ISGs) in primary RNase H2^ΔGFAP^ astrocytes 7 days post isolation, as quantified by qPCR. ISG transcript levels in RNase H2^ΔGFAP^ astrocytes were normalized to GAPDH and indicated relative to control astrocytes (*x* = 1). Error bars represent SEM, *p < 0.05, *t*-test (*n* = 3). **(B)** Representative Immunofluorescence analysis (*n* = 3) showing extensive DNA damage in RNase H2^ΔGFAP^ astrocytes 7 days post isolation, as evidenced by histone H2AX phosphorylation (γH2AX). Wild-type astrocytes treated with 1 µM Doxorubicin for 24 h served as positive control. DAPI as nuclear counterstain. **(C)** Representative flow cytometry histogram showing increased DNA damage in RNase H2^ΔGFAP^ astrocytes, compared to control astrocytes (*n* = 3). Unstained cells were used as negative control. **(D)** EdU incorporation assay (4 h pulse) revealed significantly fewer RNase H2^ΔGFAP^ astrocytes in S-Phase 7 days post isolation. Error bars represent SEM, **p < 0.01, *t*-test (*n* = 4). **(E)** Immunoblot showing the absence of apoptosis in RNase H2^ΔGFAP^ astrocytes, as evidenced by lack of PARP-1 cleavage (*n* = 2). PARP-1 is a caspase-3 substrate. Control astrocytes treated with 25 µM Doxorubicin for 24 h served as positive control, while β-Actin levels indicated equal loading. fl = full length, cl = cleaved.

**Figure 5:**
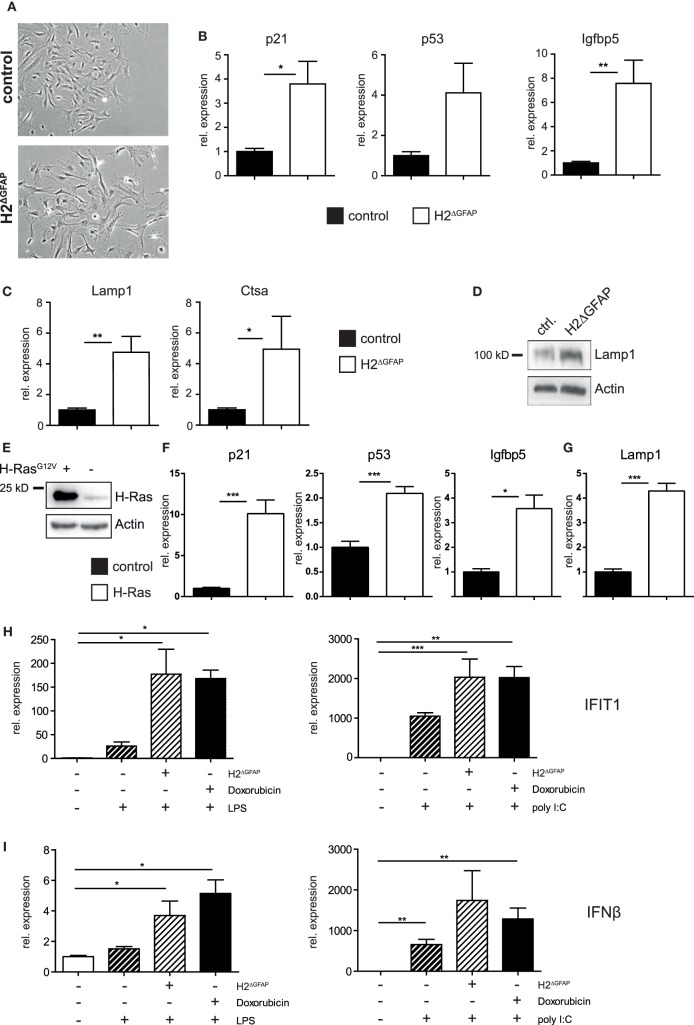
RNase H2^ΔGFAP^ astrocytes show signs of premature senescence and an amplified immune response toward bacterial and viral antigens. **(A)** RNase H2^ΔGFAP^ astrocytes displayed an enlarged, flattened morphology 7 days after isolation, indicative of premature senescence (*n* = 3). **(B)** Increased expression of senescence-associated genes in RNase H2^ΔGFAP^ astrocytes compared to control astrocytes (*x* = 1) 10 days post isolation. Transcript levels were quantified by qPCR and normalized to GAPDH expression. Error bars are SEM, *p < 0.05, **p < 0.01, *t*-test (*n* = 3). **(C,D)** Elevated transcript (Lamp1, Ctsa) and protein (Lamp1) levels of lysosomal genes suggested enlargement of the lysosomal compartment in RNase H2^ΔGFAP^ astrocytes, a hallmark of cellular senescence. Expression was assessed by qPCR **(C)** or Western Blot **(D)**, β-actin as loading control. Error bars are SEM, *p < 0.05, **p < 0.01, *t*-test (*n* = 3). **(E)** Control astrocytes were transduced with a retrovirus encoding H-Ras^G12V^ and successful transduction was confirmed by Western Blot against H-Ras. 6 days after transduction with oncogenic H-Ras, astrocytes showed mRNA upregulation of senescence-associated **(F)** as well as lysosomal genes **(G)**, as measured by qPCR. Error bars are SEM, *p < 0.05, ***p < 0.001, *t*-test (*n* = 3). **(H,I)** Isolated murine astrocytes were primed by DNA damage and then challenged with bacterial (LPS, left) or viral (polyI:C, right) antigens. DNA damage was provoked either by treatment with Doxorubicin or RNase H2-deficiency (H2^ΔGFAP^). Activation of the innate immune system was assessed by IFIT1 **(H)** and IFNβ **(I)** qPCR. Increase in mRNA expression is indicated relative to unchallenged control astrocytes (*x* = 1). Astrocytes were treated 7 days post isolation. Error bars represent SEM, *p < 0.05, **p < 0.01, ***p < 0.001, *t*-test (*n* ≥ 3).

Skin fibroblasts from AGS patients bearing RNase H2 mutations displayed signs of DNA damage, culminating in the development of premature senescence in diseased fibroblasts (28). Given the established link between DNA damage and type I interferon expression, it has been hypothesized that in fact DNA damage might represent the prime trigger of the pathogenic type I interferon production in AGS (12, 28, 29). We sought to investigate whether RNase H2^ΔGFAP^ astrocytes would recapitulate some of the features of AGS patient cells. We, therefore isolated astrocytes from neonatal RNase H2^ΔGFAP^ and control brains, with primary cell cultures consistently containing >90% GFAP-positive murine astrocytes (a representative astrocyte preparation is shown in Figure S4D in Supplementary Material). As shown above, cultured astrocytes from RNase H2^ΔGFAP^ mice were devoid of RNase H2 (Figures 2C,D). In contrast to RNase H2^ΔGFAP^ brains, which failed to show significant ISG upregulation (Figure 3C), we observed a robust interferon signature in cultured RNase H2^ΔGFAP^ astrocytes (Figure 4A). Importantly, assessment of cerebral ISG expression was carried out at the same developmental stage as RNase H2^ΔGFAP^ astrocyte isolation (P3/4 pups). Using flow cytometry and immunofluorescence, we monitored phosphorylation of histone H2AX, a common marker for DNA double-strand breaks, and detected extensive DNA damage in RNase H2^ΔGFAP^ astrocytes (Figures 4B,C). In line with increased DNA damage, RNase H2^ΔGFAP^ astrocytes showed a marked reduction in their proliferative capacity, as assessed by EdU incorporation (Figure 4D). Western blot analysis revealed that RNase H2^ΔGFAP^ astrocytes did not exhibit caspase-mediated PARP-1 cleavage, indicating that RNase H2-deficient astrocytes did not undergo apoptosis (Figure 4E). In contrast, RNase H2^ΔGFAP^ astrocytes developed an enlarged and flattened morphology, which is consistent with cellular senescence (Figure 5A). In keeping with this, transcript levels of several senescence-associated genes were upregulated in RNase H2^ΔGFAP^ astrocytes (Figure 5B). A hallmark of senescent cells is the enlargement of their lysosomal compartment, which is typically assessed by measuring activity of acidic β-galactosidase (30). However, high background levels of acidic β-galactosidase already in control astrocytes precluded a detailed analysis of this marker (data not shown). Instead, we observed increased mRNA and protein levels of the lysosomal proteins LAMP1 and Cathepsin A in RNase H2^ΔGFAP^ astrocytes, which likewise suggested elevated lysosome biogenesis in the absence of RNase H2 (Figures 5C,D). Importantly, control astrocytes that had been driven into oncogene-induced senescence (OIS) by retroviral transduction with oncogenic H-Ras showed comparible upregulation of senescence-associated genes (Figures 5E–G).

It has been hypothesized that AGS might require an environmental trigger for manifestation (see above). To test this hypothesis at the cellular level, we challenged isolated astrocytes “primed by DNA damage” with bacterial (LPS) and viral (poly I:C) antigens. DNA damage was inflicted either by treatment with the chemotherapeutic drug doxorubicin or RNase H2 loss (in RNase H2^ΔGFAP^ astrocytes). Importantly, when isolated astrocytes were primed by DNA damage prior to LPS or poly I:C treatment, enhanced activation of the type I interferon axis was observed (Figures 5H,I). Taken together, cultured murine astrocytes lacking RNase H2 exhibited increased ISG expression, extensive DNA damage, and signs of cellular senescence, all of which has previously been reported for mutant RNase H2 patient cells (28). Moreover, RNase H2-deficient astrocytes adapted a primed state that further amplified the innate immune response elicited by bacterial or viral antigens.

## Discussion

Aicardi–Goutières syndrome is a severe autoimmune childhood disorder resulting in profound physical and intellectual disabilities. AGS is of genetic origin and mainly affects the brain, although extraneurological symptoms such as chilblain lesions of the skin are frequent (2). The disease trigger is most likely a chronic overactivation of the type I interferon system, which is supported by the AGS-like phenotype of transgenic mice overexpressing interferon α in the CNS (7). All genes associated with AGS are involved either in nucleic acid metabolism or detection, with mutations in the endoribonuclease RNase H2 accounting for >50% of cases (2). In addition, gene variants in the AGS genes *TREX1* and *RNASEH2* have also been associated with SLE, underlining the considerable phenotypic overlap between the two disease entities (28, 31). RNase H2 has two distinct enzymatic activities. While it can cleave the RNA moiety of an RNA/DNA hybrid (containing >4 consecutive ribonucleotides), it is also capable of initiating the excision of single ribonucleotides embedded in a DNA duplex (32). The latter enzyme activity appears to be the main physiological function of RNase H2, as mouse embryos deficient for RNase H2 strongly accumulate genome-embedded ribonucleotides leading to widespread DNA damage (12). In this context, replicative DNA polymerases have been shown to be much more promiscuous as previously anticipated, resulting in the establishment of misincorporated ribonucleotides as the most frequent naturally occurring DNA lesions (12, 33). In line with this, RNase H2 expression is particularly strong in tissues with a high proliferative turnover, such as gut, skin, testis, and developing brain (12). Given this strong link between RNase H2 expression and proliferation, we were surprised by the high abundance of RNase H2 in postmitotic neurons. A possible explanation for the unexpectedly strong expression in postmitotic neurons could be a function of RNase H2 independent of DNA replication such as R-loop resolution during transcription (34) or RNA/DNA hybrid degradation during double strand break repair (35). However, this potential alternative function of RNase H2 in the brain is apparently not crucial for neuronal homeostasis, as neuronal RNase H2 ablation did not result in an obvious phenotype (this study). RNase H2 knockout mice die *in utero* as a consequence of a p53-mediated growth arrest starting around gastrulation (11, 12). As due to their early embryonic lethality RNase H2 null mice preclude detailed analysis of AGS pathology, we generated conditional knockout mice lacking RNase H2 in the brain, the most affected organ in AGS. To that end, we intercrossed floxed RNase H2B mice to animals expressing Cre under the human GFAP promoter, thereby targeting essentially the same cell types as in the seminal study on brain-specific interferon α overexpression (7). In RNase H2^ΔGFAP^ mice, RNase H2 ablation was not only achieved in astrocytes but also in almost all cortical and hippocampal neurons. The efficient deletion of RNase H2 in neurons of RNase H2^ΔGFAP^ mice somewhat surprised us, although recombination activity outside astrocytes has previously been reported for this Cre line (27). Consequently, the RNase H2 deletion pattern observed in RNase H2^ΔGFAP^ mice strongly resembled a second, neuron-specific RNase H2 mutant mouse line we generated in this study (RNase H2^ΔEmx1^). Despite a widespread absence of RNase H2 in astrocytes and neurons, RNase H2^ΔGFAP^ mice did not develop clinical signs resembling AGS, such as neuroinflammation, brain calcifications, or leukodystrophy. The lack of an overt phenotype in RNase H2^ΔGFAP^ mice is remarkable, given (1) the essential role of RNase H2 in prenatal development and (2) the severe consequences of only hypomorphic RNase H2 mutations in AGS patients. This discrepancy between mouse model and human disease does not only hold true for RNase H2, but also for other AGS-associated genes. In a nutshell, all AGS mouse models described so far fail to recapitulate the strong CNS involvement of the human disease. The reason for this striking difference in disease course between the two species is not known at present and can only be speculated on. At least for RNase H2, however, our study might offer a possible explanation for this apparent conundrum. Whereas RNase H2^ΔGFAP^ mice did not exhibit AGS-related disease signs *in vivo*, RNase H2^ΔGFAP^ astrocytes isolated from the same animals showed cellular defects akin to AGS patient cells, including DNA damage, premature senescence, and increased ISG expression. In this context, it is important to note that RNase H2^ΔGFAP^ astrocytes were cultured under mitogenic conditions, which is in stark contrast to the largely postmitotic nature of resident neurons and astrocytes in RNase H2^ΔGFAP^ brains. It is therefore plausible that active proliferation is a prerequisite for the development of cellular features reminiscent of AGS. In support of this, recent reports have shed light on the role of micronuclei in activating the innate immune system upon DNA damage, which in two studies also included DNA damage caused by RNase H2 loss (36–38). Importantly, generation of these immunogenic micronuclei was strongly dependent on cellular proliferation (36–38). This reliance on proliferation could explain why humans with only hypomorphic RNase H2 mutations develop AGS, whereas mice carrying the same patient mutations (16, 17) or even completely lack RNase H2 in large parts of the brain (this study) do not show AGS-like features: Due to the larger size of the human brain neurogenesis is faster and involves more cell divisions than in the murine brain (39). It is, therefore conceivable that the degree of neuronal proliferation is just not sufficient for the establishment of disease symptoms in the much smaller murine brain. However, further experiments are clearly needed to test this hypothesis. In aggregate, primary RNase H2^ΔGFAP^ astrocytes can serve as a valid cellular model of AGS, since they (1) display similar defects as RNase H2 mutant AGS patient cells and (2) constitute the very cell type responsible for pathogenic interferon α production in AGS individuals. In addition, DNA damage renders RNase H2-deficient astrocytes more sensitive toward foreign antigens, which could help explain the phenotypic variability among AGS patients bearing the same mutation and also why AGS sometimes seems to require an exogenous trigger in order to manifest.

## Ethics Statement

All statements regarding the mouse work have been included in the Section “Materials and Methods” of the manuscript.

## Author Contributions

KB, MD, and BR designed the study. KB, MD, TR, LB, LG, SH, KK, CB, and MG performed experiments and analyzed the data. AW, HF, VG-D, MHA, and BR planned the projects and supervised the experiments. KB, MD, and BR wrote the manuscript.

## Conflict of Interest Statement

The authors declare that the research was conducted in the absence of any commercial or financial relationships that could be construed as a potential conflict of interest.
