# Supplementary material for: RNase H2 Loss in Murine Astrocytes Results in Cellular Defects Reminiscent of Nucleic Acid-Mediated Autoinflammation
Source: Front Immunol. 2018 Mar 29;9:587. doi: 10.3389/fimmu.2018.00587 (PMC5890188; doi:10.3389/fimmu.2018.00587)
Supplement: Supplementary file 2 [file presentation_1.PDF]

Suppl. Fig. 1:

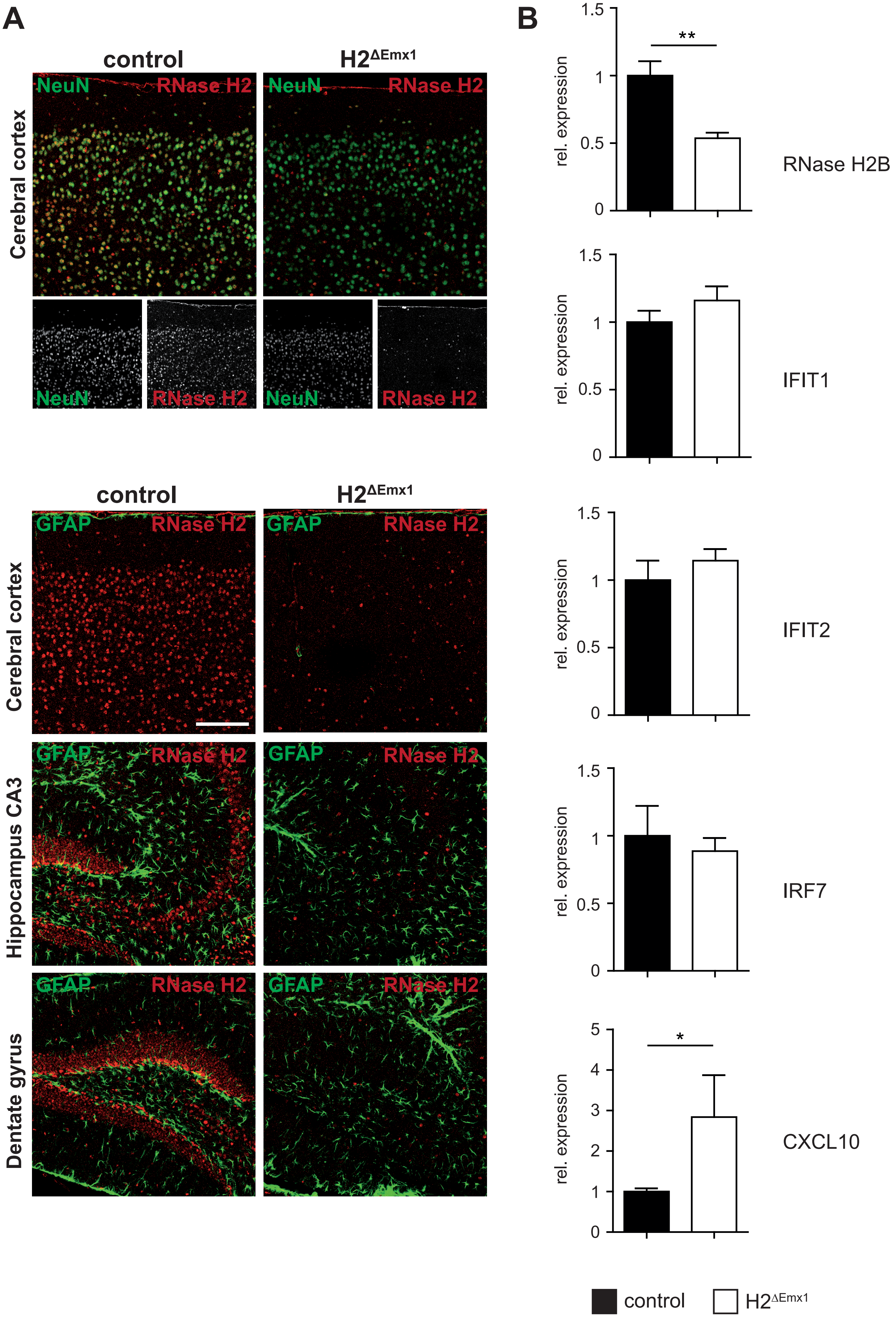
A
control
H2ΔEmx1
Cerebral cortex
NeuN
RNase H2
control
H2ΔEmx1
Cerebral cortex
Hippocampus CA3
Dentate gyrus
GFAP
RNase H2
B
rel. expression
RNase H2B
IFIT1
IFIT2
IRF7
CXCL10
**
*
control
H2ΔEmx1

Suppl. Fig. 2:

A

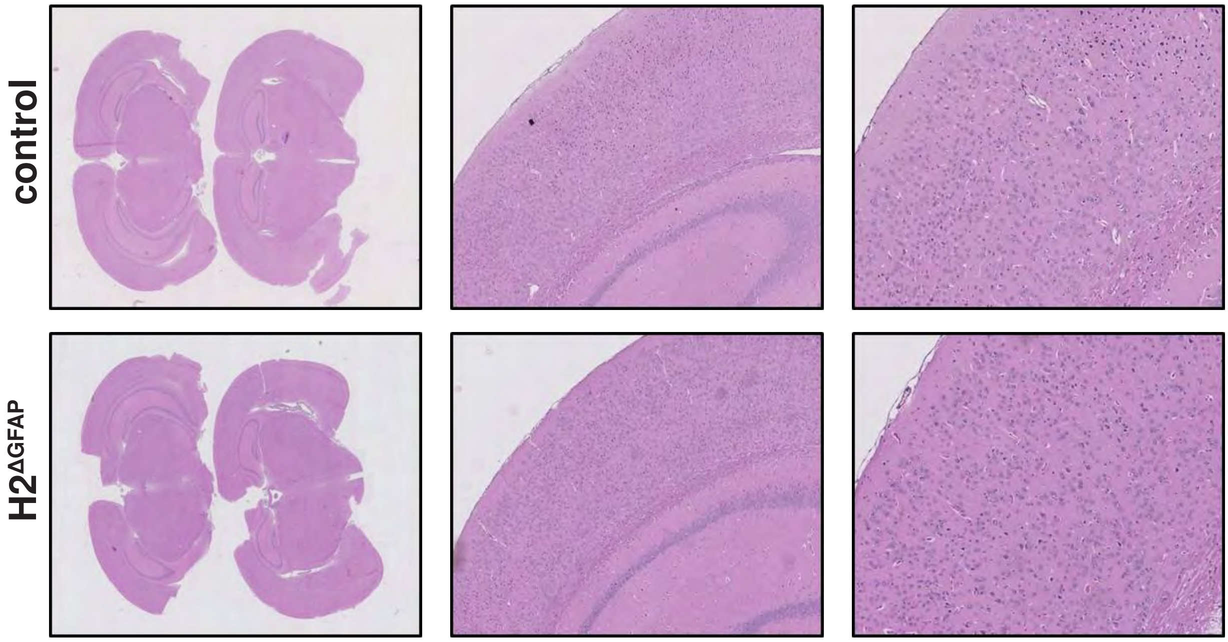

B

Test	Age (weeks)	Result
**Open Field**	8	Reduced rearing in females
**SHIRPA**	9	No differences
**Grip strength**	9	No differences
**ASR/PPI**	10	No differences
**Rotarod**	13	No differences
**Hotplate**	13	Subtle effect (slightly faster second reaction)
**ABR**	14	No differences
**Balance Beam**	52	Subtle findings for single beams (more traversing time beam 1, more stops of females on beams 1 and 2)
**Y-Maze**	53	Increased same arm entries in males
**Social Discrimination**	54	Subtle finding (trend for reduced social affinity in males)
**Vertical Pole**	57	No significant differences
**Beam ladder**	60	No significant differences

ASR/PPI: Acoustic startle response/Pre-pulse inhibition
ABR: Auditory brain stem response

Suppl. Fig. 3:

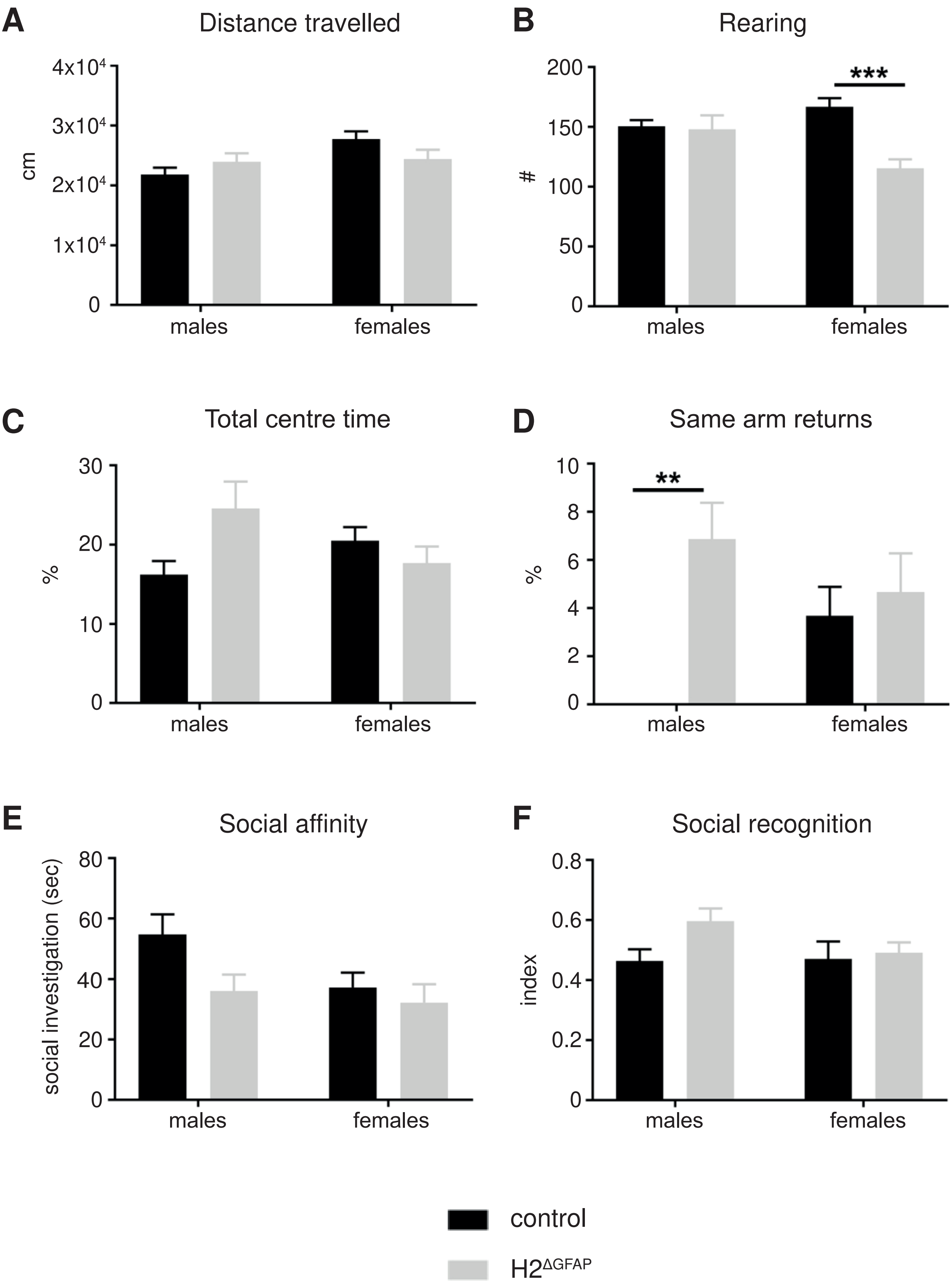
A
Distance travelled
cm
4x10⁴
3x10⁴
2x10⁴
1x10⁴
0
males
females
B
Rearing
#
200
150
100
50
0
***
males
females
C
Total centre time
%
30
20
10
0
males
females
D
Same arm returns
%
10
8
6
4
2
0
**
males
females
E
Social affinity
social investigation (sec)
80
60
40
20
0
males
females
F
Social recognition
index
0.8
0.6
0.4
0.2
0
males
females
control
H2ΔGFAP

Suppl. Fig. 4 :

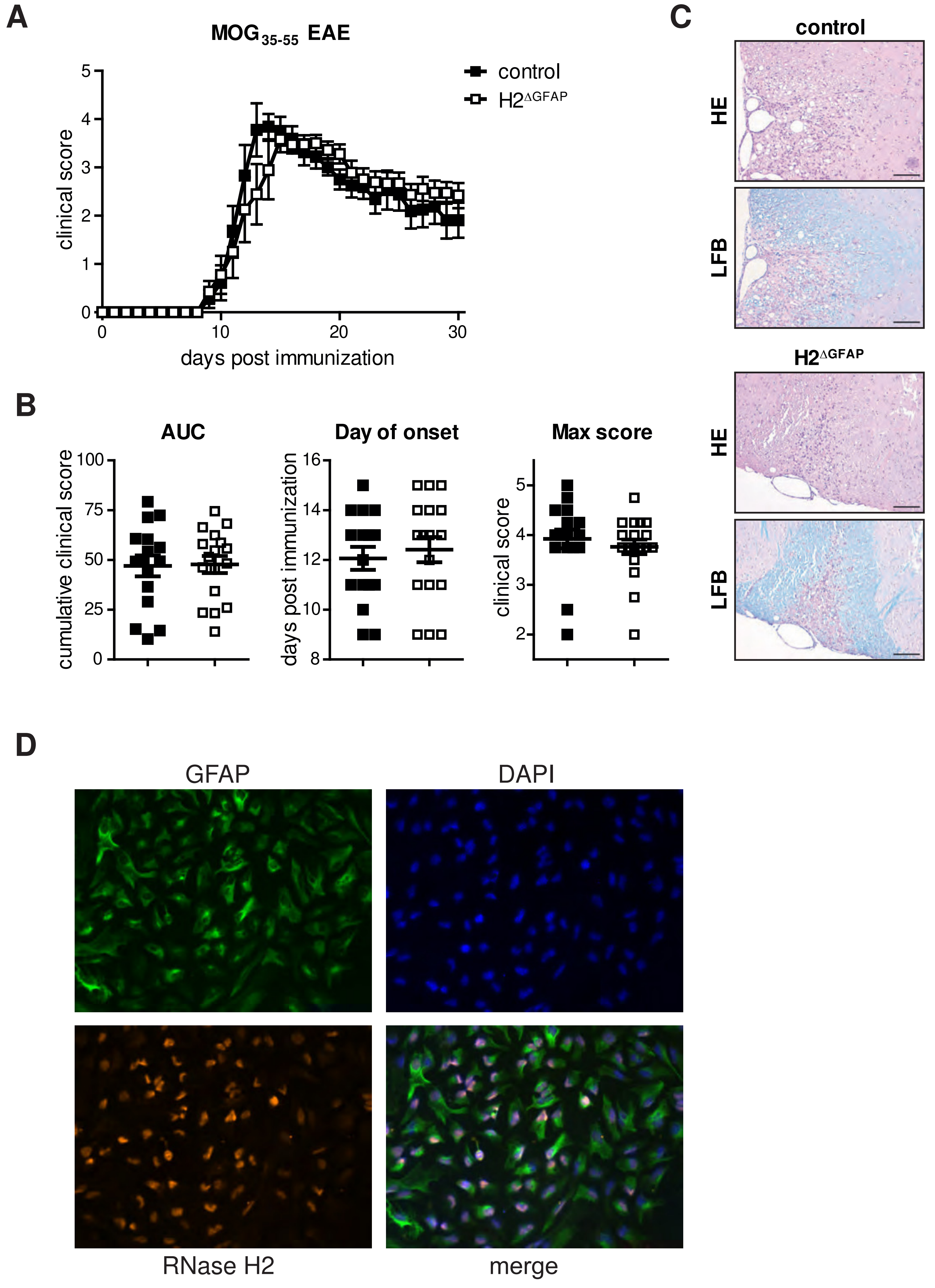
A
MOG35-55 EAE
control
H2ΔGFAP
clinical score
days post immunization
0
1
2
3
4
5
10
20
30
B
AUC
cumulative clinical score
0
25
50
75
100
Day of onset
days post immunization
8
10
12
14
16
Max score
clinical score
2
3
4
5
C
control
HE
LFB
H2ΔGFAP
HE
LFB
D
GFAP
DAPI
RNase H2
merge

Supplementary Figure legends

Suppl. Figure 1: Basal analysis of RNase H2$^{\Delta Emx1}$ mice.

(A) Top: Absence of RNase H2 in the majority of neurons in the cerebral cortex of RNase H2$^{\Delta Emx1}$ mice (age 6 weeks). Neurons were co-stained with the neuronal marker NeuN. Bottom: Lack of RNase H2 in different brain regions of RNase H2$^{\Delta Emx1}$ mice. GFAP immunohistochemistry revealed no apparent astrogliosis. Astrogliosis would present as increased expression of GFAP, cellular hypertrophy with changes of astrocyte morphology or proliferation of astrocytes. (B) Comparable ISG expression in cortices of RNase H2$^{\Delta Emx1}$ mice, as quantified by qPCR. mRNA levels were normalized to GAPDH and indicated relative to control cortices (x=1). CXCL10 was slightly, but signficantly upregulated. In line with neuronal RNase H2 loss, RNase H2B transcript levels were reduced in RNase H2$^{\Delta Emx1}$ cortices. Error bars are SEM, * $p < 0.05$, ** $p < 0.01$, t-test (n=3).

Suppl. Figure 2: No overt structural, neurological or behavioral changes in RNase H2$^{\Delta GFAP}$ mice.

A) HE staining of RNase H2$^{\Delta GFAP}$ brains showed no morphological differences between genotypes (n=3) at 8 months of age. B) Tabular summary of the neurological and behavioral analyses. Only subtle, sporadic and mostly sex-specific changes with no apparent relation to AGS were observed in RNase H2$^{\Delta GFAP}$ mice. For detailed reports see Suppl. Fig. 3 (behaviour screen) and Suppl. Data. (neurology screen).

Suppl. Figure 3: Detailed behavioral characterization of RNase H2$^{\Delta GFAP}$ mice.

Open Field test (A-C). Spontaneous locomotor activity in a novel environment as measured by total distance travelled (A) at the age of 8 weeks yielded no difference in forward locomotor activity, but a significantly reduced rearing activity (B) in female RNase H2$^{\Delta GFAP}$ mice only (Two-way ANOVA with Sidak's post-hoc test corrected for multiple comparisons; genotype x sex interaction: $F(1,35) = 6.494$, $p < 0.05$; adjusted p-value post-hoc test females: $p < 0.001$). RNase H2-deficiency in the brain affected anxiety-related behavior as measured by the time spent in the centre of the Open Field (C) only subtly and in opposite directions in the two sexes (genotype x sex interaction: $F(1,35) = 4.425$, $p < 0.05$; post-hoc tests n.s.). Spontaneous alternation in the Y-Maze did not reveal any difference in working memory, but an increase of same arm returns that was only significant in male RNase H2$^{\Delta GFAP}$ mice (D), suggesting an increase in repetitive behavior mice (genotype x sex interaction: $F(1,29) = 4.498$, $p < 0.05$; adjusted p-value post-hoc test males: $p < 0.01$). Social discrimination test (E,F). Analysis of social recognition memory demonstrated a trend towards reduction in social affinity (E) that just missed significance (genotype effect: $F(1,29) = 4.165$, $p = 0.0505$), and no difference in the social recognition index (F). There were no genotype effects in sensorimotor gating behavior as measured by prepulse inhibition of the acoustic startle reflex (data not shown).
Several tests for basic neurological function did not reveal clear differences either. There were subtle significant changes in single parameters but no clear indication for neurological dysfunctions in the mice tested at the age of 9-14 weeks (see supplemental Neurology data). To exclude a later onset of symptoms, mice were analyzed at the age of 12-14 months again with additional motor tests but again showing only single alterations (see supplemental Neurology data).

Suppl. Fig. 4: No exacerbation of CNS autoimmunity in RNase H2$^{\Delta GFAP}$ mice.

A) Following immunization with myelin oligodendrocyte glycoprotein peptide (MOG35-55), RNase H2$^{\Delta GFAP}$ and control mice developed similar clinical signs of ascending paralysis. Disease course of control (n=9) versus RNase H2$^{\Delta GFAP}$ (n=10) mice after immunization with MOG/CFA/Ptx. Graph is representative of 2 independent experiments. B) Analysis of clinical

parameters, including disease onset, maximum and cumulative clinical score, revealed no differences between RNase H2$^{\Delta GFAP}$ and control mice. EAE disease parameters depicting the area under the curve (AUC), day of onset and maximum clinical score of EAE-immunized control (n=16) and RNase H2$^{\Delta GFAP}$ (n=18) mice. Data is summarized from both independent experiments. C) Similar clinical manifestation was further confirmed by the degree of demyelination in spinal cords of RNase H2$^{\Delta GFAP}$ versus control mice. Hematoxylin/Eosin (HE) and Luxol fast blue (LFB) stainings of spinal cord sections from EAE-afflicted control and RNase H2$^{\Delta GFAP}$ mice. Note the lesion areas as manifested by dense leukocyte infiltration (HE) co-localizing with apparent demyelination (LFB). Pictures are representative for 4 mice with similar disease scores examined in each group. Scale bar = 100µm. D) Primary cultures generated from P3/4 brains consistently contained > 90% of GFAP-positive murine astrocytes. A representative astrocyte preparation from an RNase H2-proficient control brain is shown. Cells were counter-stained with DAPI and rabbit anti-RNase H2 antiserum.

Supplementary tables

A Nucleotide sequences of qPCR primers used in the study

Gene	forward primer	reverse primer
Ifit1	GAACCCATTGGGGATGCACAACCT	CTTGTCCAGGTAGATCTGGGCTTCT
Ifit2	ATGAGTTTCAGAACAGTGAGTTTAA	AACTGGCCCATGTGATAGTAGACCC
Irf7	ATGCACAGATCTTCAAGGCCTGGGC	GTGCTGTGGAGTGCACAGCGGAAGT
Cxcl10	GCCGTCATTTTCTGCCTCA	CGTCCTTGCGAGAGGGATC
Cdkn1a (p21)	CCTGGTGATGTCCGACCTGTT	GGGGAATCTTCAGGCCGCTC
Trp53	CTAGCATTCAGGCCCTCATC	TCCGACTGTGACTCCTCCAT
Igfbp5	CGCGGGGTTTGCCTCAACGA	CTGCGGCAGGGGCCTTGTTC
Lamp1	TAATGGCCAGCTTCTCTGCCTCCTT	AGGCTGGGGTCAGAAACATTTTCTT
Ctsa	GACTCCAAGCACTTCCACTACTGGT	CTGGCTGGATCAGAAAGGGGCCGTG
Gapdh	TTCACCACCATGGAGAAGGC	GGCATCGACTGTGGTCATGA
Rnaseh2b	AGGTTTCCAGGGACAAGGAAGAGGA	GTCAATGAAGCTGGAGGTTCTGGAAG

B Dilutions of antibodies used in the study

Antibody	Application	Dilution 1st AB	2nd AB	Dilution 2nd AB
α-mouse RNase H2 holoenzyme	IHC, IF	both 1:250	α-rabbit	1:5000
α-NeuN	IHC	1:1500	α-mouse	1:500
α-GFAP	IHC	1:500	α-mouse	1:500
α-Iba-1	IHC	1:500	α-rabbit	1:500
α-Ki-67	IHC	1:500	α-rat	1:500
α-RNase H2A	WB	1:1000	α-rabbit	1:5000
α-PARP1	WB	1:1000	α-rabbit	1:5000
α-γH2AX	IF, FACS	both 1:75	α-rabbit	1:1000
α-LAMP1	WB	1:1000	α-mouse	1:5000
α-H-Ras	WB	1:500	α-mouse	1:5000

IHC: Immunohistochemistry (frozen sections)
IF: Immunofluorescence
WB: Western Blotting
FACS: Flow Cytometry
AB: Antibody
